# Association of platelet count and mean platelet volume (MPV) index with types of stroke

**DOI:** 10.22088/cjim.11.4.398

**Published:** 2020

**Authors:** Bahram Zarmehri, Behzad shahi, Shaghayegh Rahmani, Fatemeh Dehghan Tafti, Mahdi Foroughian

**Affiliations:** 1 Department of Emergency Medicine, Faculty of Medicine, Mashhad University of Medical Sciences, Mashhad, Iran; 2 Department of Anesthesiology, Mashhad University of Medical Sciences, Mashhad, Iran

**Keywords:** Stroke, Platelet count, Mean platelet volume index

## Abstract

**Background:**

Stroke is known to be the third most prominent cause of death in the developing countries and the most common debilitating neurologic disease. This study aimed to investigate the association of platelet count (PC) and mean platelet volume (MPV) index with various stroke types.

**Methods:**

This cross-sectional study was carried out on patients over the age of 18 years who presented with signs and symptoms of the first acute stroke. Exclusion criteria were underlying chronic liver or renal disease and the time more than 6 hours from symptom initiation, hematological and infectious disorders in patients. After recording of demographic data, a complete blood cell count (CBC) test was performed.

**Results:**

From 150 patients, who enrolled in the study, 54.7% of patients were males. The initial brain CT scan was normal in 13 (8.7%) patients and showed evidence of brain infarction and intracranial hemorrhage in 84 (56%) and 53 (35.3%) patients respectively. Patients with intracranial hemorrhage had significantly higher mean of MPV index than the patients with normal brain-CT scan and patients with evidence of brain infarction (p<0.001).

**Conclusion:**

The MVP index can be a predictor of the type of hemorrhagic or ischemic finding in emergency CT scan in stroke patients. This relationship may help to better understand the physiopathologic role of platelets in the development of stroke (hemorrhagic or ischemic), but will not replace cerebral computed tomography to diagnose the type of stroke, or it may not initiate treatment for hemorrhagic stroke.

Stroke is known to be the third leading cause of death worldwide and is rapidly becoming a global health problem. It may become a serious crisis in the future due to demographic changes such as population aging (1, 2 and 3). Atherothrombosis and thromboembolism are the main causes of ischemic stroke. Platelets are important components of thrombosis and play a critical role in the thrombosis, which may be activated by platelet rupture, inflammation, and other conditions (4, 5). Activated platelets initiate the formation of hemostatic plug and trigger the coagulation pathway (6, 7). Platelet indices are considered for the assessment of platelet activity. Platelet count (PC), mean platelet volume (MPV), and platelet distribution width (PDW) are some of platelet indices. Generally, it is known that larger platelets contain more granules than smaller platelets and have higher levels of platelet aggregation markers such as β-thromboglobulin and thromboxane B2. Also, platelets with larger volume have higher metabolic and enzymatic activity than platelets with lower volume. In some studies, increased MPV was indicated to be associated with increased platelet activity and therefore, may be related to the severity and prognosis of stroke; the higher MPV may result in a worse outcome (8-14).

It has also been documented that the platelet dysfunction is attributed to an increased risk of intracranial hemorrhage, and the PC on the first day is an important factor in predicting mortality rate (15, 16). Fasting blood glucose and lipid testing and blood count formula and ECG are mandatory in all of these patients (17-19).Brain CT scans are required in every stroke patient, but if the stroke occurs within the first 12 hours, the picture of the stroke may not yet be detected in the brain CT scan (20). Therefore, not having a stroke on a patient's CT scan does not mean that the patient is not having a stroke (20-21). More accurate imaging techniques such as MRI, CT angiography are recommended (22). Considering the limited studies on the association of PC and MPV with type of stroke, the present study examined the association of PC and MPV with the type of stroke in the patients referring to the emergency department(ED) of Ghaem Hospital, Mashhad, Iran in 2018, with focal neurologic signs.

## Methods

The Research Ethics Committee of Mashhad University of Medical Sciences has endorsed this descriptive cross-sectional research (IR.MUMS.FM.REC.1396.98), and this study was conducted between May 2018 and January 2019.

**Sample size: **According to α=0.05 and β=0.20, and a study in Iran by Alidaei et al. (23), the sample size was estimated at 50 patients in each group. Data from the demographic and paraclinical profiles of the patients were coded into the software.

**Inclusion and exclusion criteria: **The inclusion criteria were the patients referred with the signs and symptoms of the acute stroke for the first time. Patients with all forms of hemorrhagic stroke (subarachnoid, subdural, epidural, intraparenchymal, and intraventricular hemorrhages), or having a previous stroke attack history, transient ischemic attack (TIA), or having any underlying disease such as malignancies, connective tissue disorders, vasculitis, autoimmune diseases like rheumatoid arthritis, systemic lupus erythematosus, and chronic renal and hepatic insufficiency, pancreatitis, and patients who had organ transplantation history, or other immunosuppressive conditions, a history of previous thrombosis, hemoglobinopathies, and patients with fever at presentation as MPV may have been affected were also excluded (24). 

**Data collection tools: **Patient demographic data, including age, gender, and baseline symptoms, were recorded in the pre-prepared checklist.

**Patients' Choice:** Patients were followed-up consecutively and referred to the emergency clinic

**Study methodology: **On admission to the emergency department, CBC test was carried out for the patients using five cc blood sample taken from the brachial vein. In the next step, an axial brain CT scan without contrast was performed, and the patients were partitioned into 3 groups of normal, hemorrhagic stroke and ischemic stroke based on the brain CT scan. Finally, the MPV level was compared with the neuroimaging findings.

**Statistical analysis: **Data were analyzed by SPSS Version 16 software using inferential statistics (independent t-test, Mann-Whitney, and univariate and multivariate regression analysis) and were reported using descriptive statistics (as well as mean and standard deviation and percentage). The level of significance was considered as p<0.05.

**Ethical considerations**

• Respecting for and observing the rights of patients in the research community

• Not mentioning the names of the patients on the checklist

• Ensuring the confidentiality of all information obtained from the research community

• Imposing no cost on patients

## Results

This study examined 150 patients, 82 (54.7%) males, and 68 (45.3%) females. An initial brain CT scan was normal in 13(8.7%) patients and showed evidence of brain infarction, and intracranial hemorrhage in 84 (56%) and 53 (35.3%) patients respectively. The mean PC in all patients was 219±64 thousand and the mean MPV index was 10.1±1 fL. The mean PC was not significantly different in three groups (P=0.357). Table 1 compares the mean PC based on the brain CT scan of the patients.

**Table 1:** Comparison of mean platelet count based on the brain CT scan

**CT scan findings**	^1^ PC * 100,000	**P -value**
Normal	218±121	0.357
Ischemic stroke	225±56	
Hemorrhagic stroke	202±64	

^1 Platelet count^

The mean of MPV index was significantly greater in patients with intracranial hemorrhage rather than patients with normal brain CT scan or patients with evidence of brain infarction (P=0.001). Pair wise comparison of mean of MPV index between the groups revealed that this index was not significantly different between the patients with normal brain CT-scan and infarction (P=0.779), this index was significantly different between the patients with normal brain CT scan and evidence of hemorrhage (P=0.001), and the mean of MPV index was significantly different between the patients with infarction and evidence of hemorrhage (P=0.001). Table 2 compares the mean MPV index based on the brain CT scan of the patients.

**Table 2:** Comparison of mean MPV index based on the brain CT scan

**CT scan findings**	**Mean MPV index** ** (fL)**	**P-value**
Normal	9.5±0.54	0.001
Ischemic stroke	9.3±0.68	
Hemorrhagic stroke	11.2±1.6	

The mean MPV index was significantly higher in the male patients with hemorrhagic stroke rather than the two other groups (P=0.008). Pair wise comparison of the mean MPV index between groups showed that the mean MPV had no significant difference between the male patients of two groups with normal CT scan and with ischemic stroke (P=0.654), but this index was significantly different between the male patients in the two groups of normal CT and the hemorrhagic stroke (P=0.009). Also, pair wise comparison of the mean MPV index between groups exhibited that the MPV index had significant difference between the male patients in the two groups with hemorrhagic and ischemic stroke (P=0.001). The MPV index in female patients with hemorrhagic stroke was higher than the patients with normal brain CT scan and ischemic stroke, but the difference between them was not significant (P=0.310). A moderate correlation was found between the MPV index and the brain CT findings (r=0.530). In addition, a strong correlation was observed between the MPV index and the duration of hospitalization (r=0.630). The mean PC to MPV index ratio in the hemorrhagic stroke group was less than two other groups, but the difference was not statistically significant (P=0.301). Table 3 shows the comparison of the mean PC to MPV index ratio between the three groups. The regression analysis revealed that the MPV correlates independently with the findings of the brain CT scan and the duration of hospitalization. In table 4, the regression analysis explains the association of the MPV with various factors of patients with focal neurologic deficit.

**Table 3:** Comparing the mean PC to MPV index ratio based on the brain CT scan

**CT scan findings**	**Ratio**	**P-value**
Normal	26.6±14.2	0.301
Ischemic stroke	23.5±7.8	
Hemorrhagic stroke	21.5±6.5	

**Table 4:** Association between MPV and various factors according to regression analysis in patients with focal neurologic deficit

**Variables **	**B**	^2^ **SE**	**β**	**t**	**Pvalue**
Sex	-0.046	0.191	-0.016	-0.239	0.811
Brain CT scan findings	1.272	0.186	0.556	6.825	0.001
Duration of hospitalization	0.014	0.007	0.163	2.027	0.044

^2^ ^standard error^

## Discussion

It is estimated that the annual prevalence of stroke is about 15 million cases worldwide; one-third of them die, and one third suffer from permanent disability. Therefore, the cost of cerebrovascular disease is significant, and imposes a vast healthcare costs to the countries. Previous studies have shown that the risk of stroke becomes twice every decade over the age of 55 years. Furthermore, due to the improvement of healthcare facilities in the developing countries like Iran, the aged population is growing; therefore, cerebrovascular disease is going to become a major problem in these countries. The platelets play a fundamental role in blood hemostasis, and the role of ineffective platelets in the progression of atherogenesis and other clinical complications, including atherosclerosis has been documented in recent years. Based on the literature review, the factors of thrombogenesis associated with the platelet system are involved in the onset and progression of atherogenesis and plug formation (25). The present study revealed no significant difference of mean PC of patients with ischemic or hemorrhagic stroke. On the other hand, the mean MPV index in hemorrhagic stroke patients was significantly greater than those with ischemic stroke. Previous studies revealed no linear relationship between the MPV index, and the PC. The mean of MPV index may be increased in some vascular risk factors such as hypercholesteremia and diabetes, and in some vascular disorders like myocardial infarction, stroke, preeclampsia and renal artery stenosis (25). Luke et al.’s (2017) study in Turkey indicated that the MPV index was not associated with prognosis in stroke patients (24). On the other hand, the results of another study (2017) on patients with stroke showed that the mean of MPV index was significantly greater in the patients with infarction in areas surrounding foramen ovale in the brain in comparison with other patients (26). Quan (2017) in China showed that the PC to MPV index ratio is a good predictor of 90-day prognosis in patients with stroke due to large arterial atherosclerosis (27). 

In our study, the mean of MPV index was significantly higher in patients with hemorrhagic stroke than in those with ischemic stroke. It seems that comprehensive studies and a longer follow-up period are needed to examine the association of MPV with the prognosis of stroke patients. In the present study, the mean PC in patients with ischemic stroke was higher than the patients with hemorrhagic stroke, but this difference was not statistically significant. A study in China (2016) revealed that an increased risk of ischemic stroke and a decreased risk of hemorrhagic stroke were associated with increased PC (28). A study (2017) in North Carolina showed that every 30,000 reductions in platelet count at the levels less than 150,000 lead to a 12% increase in the risk of stroke in CABG patients (29). In the present study, the regression analysis confirmed the association of MPV with findings of brain CT scan and duration of hospitalization. In general, there is controversial evidence regarding the effect of platelets on the incidence and prognosis of stroke, but the latest theory is related to Chen (2016), who showed that the level of circulating platelet microparticles (PMPs) was significantly associated with the size of brain infarction. This study proposed a new theory on the role of PMPs in cerebral injury (30). In conclusion Present study may suggest the MPV index as a non-expensive predictor of brain CT scan in patients with an acute focal neurologic deficit in the emergency department; the greater the MPV index, the greater the likelihood of intracranial hemorrhage in brain CT scan.

## Limitations

This study data was not based on a general community and was conducted only in a referral hospital. Therefore, patients with mild stroke symptoms, who were not referred or admitted, were not included in the study.
